# Protein composition of wheat gluten polymer fractions determined by quantitative two-dimensional gel electrophoresis and tandem mass spectrometry

**DOI:** 10.1186/1477-5956-12-8

**Published:** 2014-02-11

**Authors:** William H Vensel, Charlene K Tanaka, Susan B Altenbach

**Affiliations:** 1 USDA-ARS, Western Regional Research Center, 800 Buchanan St, Albany, CA 94710, USA

**Keywords:** Chain-terminating gliadins, Gluten polymer, Size-exclusion chromatography, Wheat flour quality

## Abstract

**Background:**

Certain wheat gluten proteins form large protein polymers that are extractable in 0.5% SDS only after sonication. Although there is a strong relationship between the amounts of these polymers in the flour and bread-making quality, the protein components of these polymers have not been thoroughly investigated.

**Results:**

Flour proteins from the US bread wheat Butte 86 were extracted in 0.5% SDS using a two-step procedure with and without sonication. Proteins were further separated by size exclusion chromatography (SEC) into monomeric and polymeric fractions and analyzed by quantitative two-dimensional gel electrophoresis (2-DE). When proteins in select 2-DE spots were identified by tandem mass spectrometry (MS/MS), overlapping spots from the different protein fractions often yielded different identifications. Most high-molecular-weight glutenin subunits (HMW-GS) and low-molecular-weight glutenin subunits (LMW-GS) partitioned into the polymer fractions, while most gliadins were found in the monomer fractions. The exceptions were alpha, gamma and omega gliadins containing odd numbers of cysteine residues. These proteins were detected in all fractions, but comprised the largest proportion of the SDS-extractable polymer fraction. Several types of non-gluten proteins also were found in the polymer fractions, including serpins, triticins and globulins. All three types were found in the largest proportions in the SDS-extractable polymer fraction.

**Conclusions:**

This is the first study to report the accumulation of gliadins containing odd numbers of cysteine residues in the SDS-extractable glutenin polymer fraction, supporting the hypothesis that these gliadins serve as chain terminators of the polymer chains. These data make it possible to formulate hypotheses about how protein composition influences polymer size and structure and provide a foundation for future experiments aimed at determining how environment affects glutenin polymer distribution. In addition, the analysis revealed additional layers of complexity to the wheat flour proteome that should be considered when evaluating quantitative 2-DE data.

## Background

Wheat flour proteins, when mixed with water, form viscoelastic mixtures suitable for creating noodles, bread and baked goods that play a major role in human diets [1,2]. Because of the economic and nutritional importance of wheat, considerable effort has been extended to characterize flour proteins and evaluate their roles in determining functional properties that define wheat quality. These storage proteins can be categorized by their solubility in aqueous alcohols and by their ability to form polymers. The glutenins are poorly soluble in alcohols, capable of forming both intra- and inter-chain disulfide linkages and are believed to be primarily responsible for forming large polymers that contribute strength and elasticity to flour dough. The main types of glutenin proteins, the high-molecular-weight glutenin subunits (HMW-GS) of 66-88 kDa and the low-molecular-weight glutenin subunits (LMW-GS) of 32-45 kDa, are linked into polymers that range in size from about 150 kDa to over 1,500 kDa [3]. In comparison, most of the gliadins (alpha, gamma and omega) are monomeric storage proteins that are soluble in alcohols, range in size from 30-50 kDa, form only intra-chain disulfide bonds and are believed to be responsible for dough viscosity and extensibility. Some gliadins however have been found in the glutenin polymer [4]. It is believed that these gliadins contain extra cysteines that are free to form intermolecular crosslinks and it has been hypothesized that these gliadins prevent elongation of the glutenin polymers and serve as terminators of the polymer. From a biochemical point of view, these proteins are clearly gliadins and sometimes are called chain-terminating gliadins [5]. However, from a technological point of view, these same proteins function as glutenin subunits and are sometimes referred to as C- and D-type LMW-GS [6].

Although sparingly soluble in alcohol, about 80% of the glutenin polymer can be solubilized with dilute SDS [7,8]. The remaining polymers require sonication for solubilization in dilute SDS, presumably because of their greater molecular weight (MW) [9]. These polymer extracts are suitable for further separation on the basis of size by size-exclusion chromatography (SEC) [9]. Improvements in solvent systems [10] and SEC column efficiency have made SEC a rapid technique for characterizing the intact polymer without disulfide bond reduction [11]. Using 0.5% SDS with and without sonication, Gupta et al. [12] separated wheat flour proteins into SDS extractable (EPP) and SDS unextractable (UPP) protein fractions and demonstrated that the amount of polymer in the unextractable fraction liberated by sonication correlated with dough strength. They also found that the ratio of HMW-GS to LMW-GS was higher in the unextractable fraction than in the extractable fraction. To date, the protein composition of polymers in the SDS extractable and unextractable fractions have most often been determined by one-dimensional gel electrophoresis (1-DE) [10-12] or reverse-phase HPLC (RP-HPLC) [13]. Because the LMW-GS and some of the gliadins have similar molecular weights, it is difficult to distinguish them with certainty by 1-DE. Additionally, 1-DE does not allow individual LMW-GS to be distinguished. The LMW-GS and some gliadins also are incompletely resolved by RP-HPLC [8,14]. Thus, these methods do not provide detailed information about the identities of specific proteins present in the fractions. Quantitative two-dimensional gel electrophoresis (2-DE) offers a more precise way to calculate the ratios of HMW-GS to LMW-GS and allows determination of the components present in the polymer. This approach, when coupled with tandem mass spectrometry (MS/MS) identifications of individual proteins, provides a valuable tool for discovering specific proteins that may be important to the formation of glutenin polymers and flour quality. In this paper, we use quantitative 2-DE and MS/MS to provide a detailed look at the compositions of SEC fractions containing large (UPP) and small (EPP) glutenin polymers.

## Results

### SEC and 2-DE of gluten polymer

**Figure 1:**
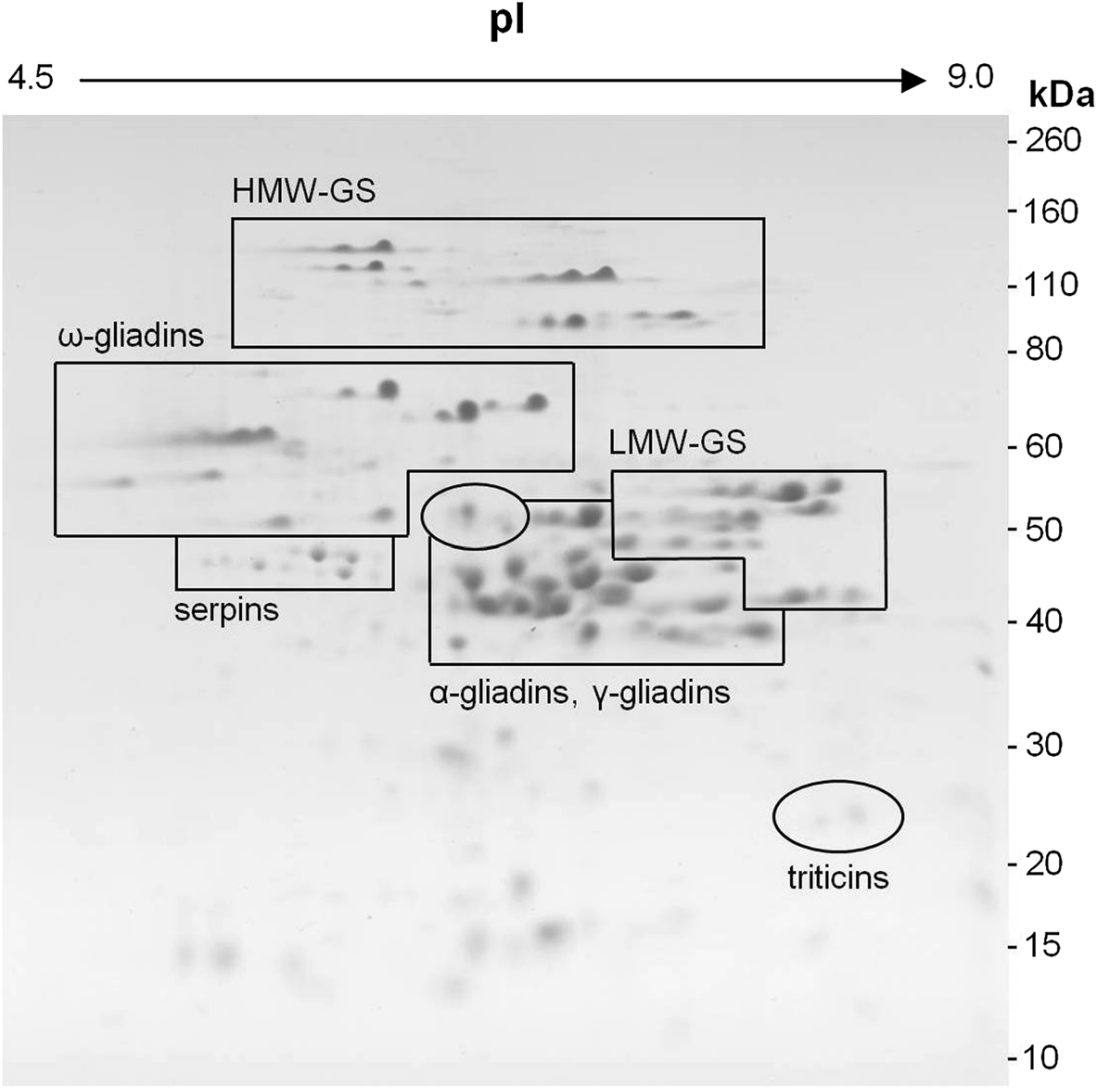
**2-DE pattern of total protein extract obtained from sonication in 0.5% SDS.** The major groups of gluten proteins are indicated. Molecular weight is shown on the vertical axis and isoelectric point is indicated by the horizontal axis.

**Figure 2:**
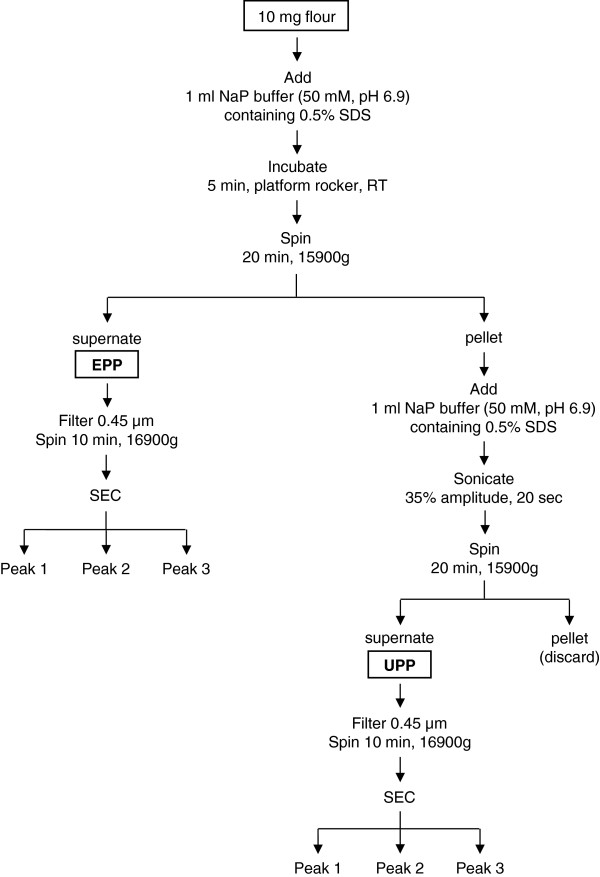
**Schema for the sequential separation of wheat flour proteins.** EPP, 0.5% SDS extractable protein fraction. UPP, 0.5% SDS unextractable, sonicated protein fraction.

Total flour protein was solubilized in 0.5% SDS with sonication as described in Materials and Methods and separated by 2-DE. The gel of that extract is presented in Figure 1 with the major protein groups indicated by the outlined regions. The HMW-GS and the omega gliadins are present in distinct areas of the gel and appear as discreet spots while the alpha and gamma gliadins and LMW-GS are more diffuse and overlap with one another. Charge trains, visible in the HMW-GS region, are often seen under the 2-DE conditions [2]. To gain needed information about the proteins that might potentially be linked in the glutenin polymer, we used 0.5% SDS to separate total flour protein into extractable polymeric protein (EPP) and unextractable polymeric protein (UPP) as outlined in Figure 2. On the basis of three replicate determinations, the average amount of protein in 100 μL of extract was 63.5 μg for the EPP and 34.4 μg for the UPP.

**Figure 3:**
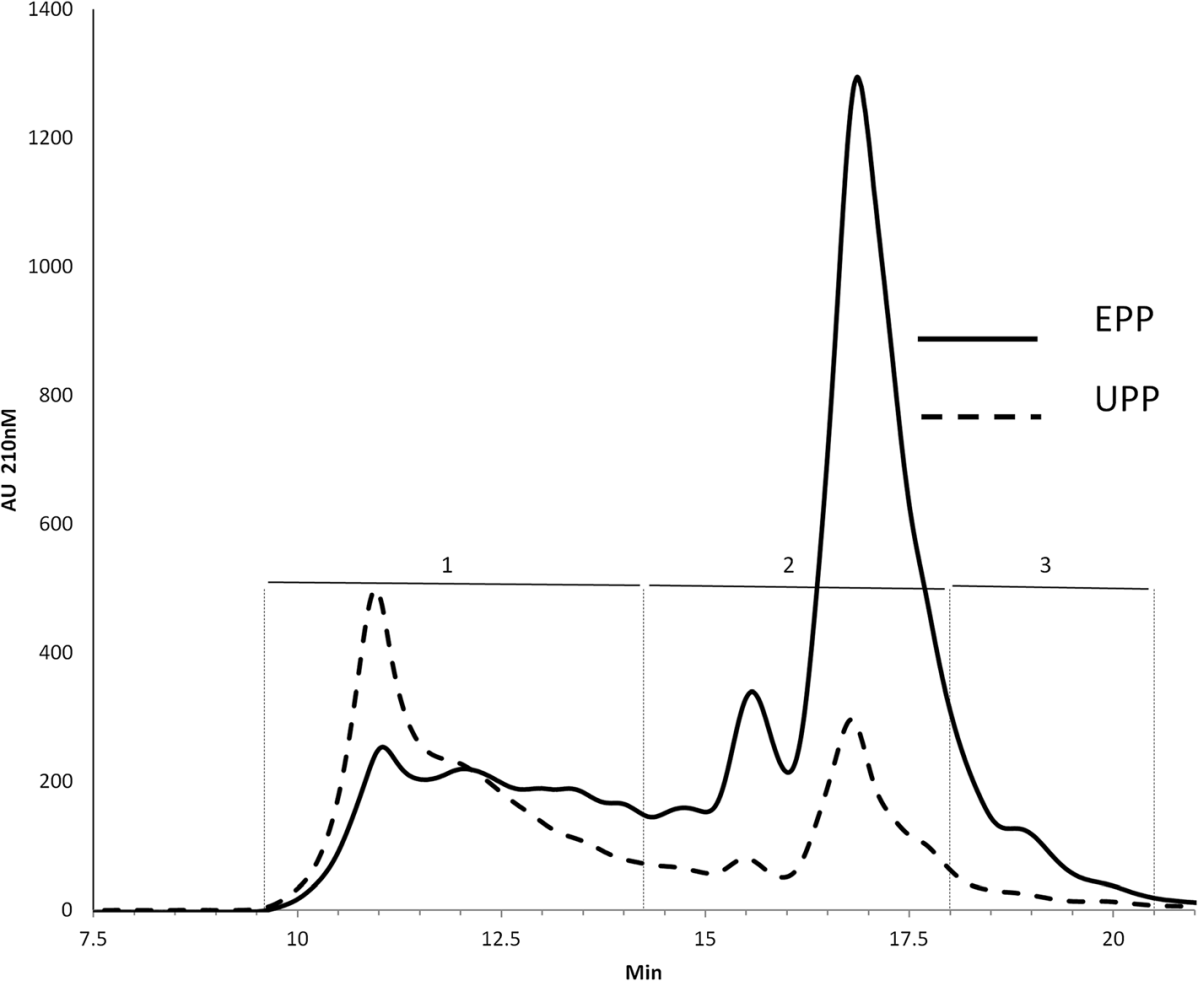
**Superimposed chromatograms from 100 μl injections of 0.5% SDS extractable glutenin polymeric protein (EPP) and sonicated unextractable polymeric protein (UPP).** Three fractions were collected for the EPP and UPP: 1) 9.6 to 14.3 min, ~2.4 ml; 2) 14.3 to 18 min, ~1.9 ml; 3) 18-20.5 min, ~1.3 ml).

**Figure 4:**
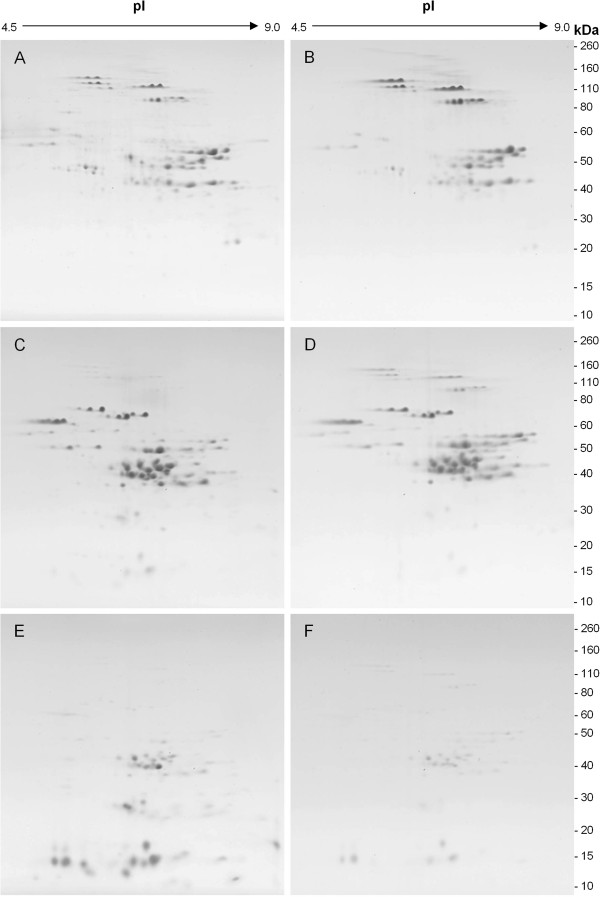
**2-D gels showing the proteins present in extractable polymeric protein (EPP) and unextractable polymeric protein (UPP) fractions. A)** EPP peak 1; **B)** UPP peak 1; **C)** EPP peak 2; **D)** UPP peak 2; **E)** EPP peak 3; **F)** UPP peak 3.

EPP and the UPP extracts were further separated by SEC (Figure 3). The glutenin polymer as well as the monomeric gliadins are soluble in 50% aqueous acetonitrile in the presence of 0.1% TFA and elute in order of size. The superimposed chromatograms can easily be divided into an early emerging peak (peak 1) and a later emerging peak (peak 2). A third fraction, referred to as peak 3, corresponds to a shoulder on the back of peak 2. The elution profile is similar to that obtained by others [3,11,15] with the polymeric components emerging in the first peak and the monomeric components in the latter half of the chromatogram. Each fraction was analyzed by 2-DE and the results in Figure 4 show that the principal proteins of peak 1 from the EPP (Figure 4A) and the UPP (Figure 4B) fractions after reduction are the HMW-GS and LMW-GS. Serpins are also visible in peak 1, particularly in the EPP fraction. In addition, peak 1 of the EPP fraction contains several proteins identified in previous studies as triticin that are not apparent in any of the other fractions. In comparison, the omega, alpha and gamma gliadins are the predominant proteins in peak 2 from the EPP (Figure 4C) and the UPP (Figure 4D) fractions. Some proteins in the LMW-GS region are also visible in both fractions. Peak 3 from both the EPP (Figure 4E) and UPP (Figure 4F) fractions contained proteins identified previously as alpha amylase/trypsin inhibitors and purinins [2]. Their presence in this fraction suggests that these proteins are not cross-linked into the polymer. Some alpha and gamma gliadins were also detected in peak 3. This is not surprising since peak 3 was collected from the tail of peak 2.

### MS/MS identification of proteins in UPP and EPP fractions

**Figure 5:**
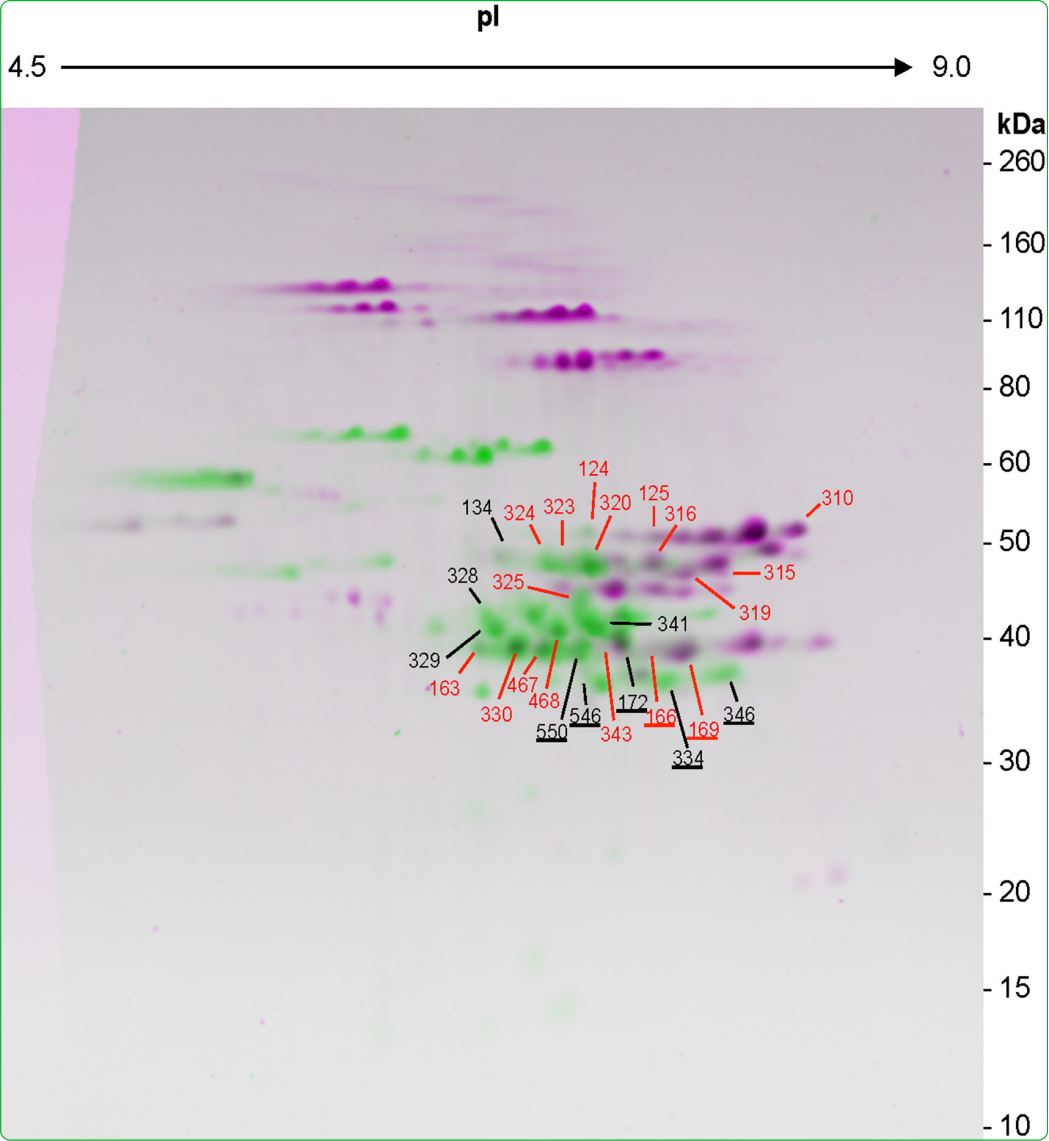
**2-DE spots that yielded different MS/MS identifications in polymer fractions than in a total protein extract.** 2-DE gel pattern of UPP peak 1 proteins (pink) was overlaid with gel pattern of UPP peak 2 proteins (green). Spots labeled with red numbers were identified as LMW-GS in the UPP peak 1 fraction but either gliadins, triticin or different LMW-GS in a total protein fraction. In at least one of the four fractions, spots labeled with underscored numbers contained chain-terminating gliadins as the predominant proteins.

Proteins in 69 of the most abundant 2-DE spots from the UPP peak 1 fraction were identified by MS/MS (Additional file 1: Table S1). Multiple proteins were identified in many spots. However, in most cases, the majority of spectra could be assigned to one protein sequence that was deemed to be the predominant protein in the spot. The predominant proteins identified in 25 2-DE spots differed between a total protein extract examined in Dupont et al. [2] and the UPP peak 1 fraction (Table 1). Eleven spots contained gliadins as the predominant proteins in the total protein fraction, but LMW-GS in the UPP peak 1 fraction (124, 163, 166, 169, 320, 323, 324, 325, 330, 467, 468) (Figure 5). In three spots, different types of LMW-GS (i-type, s-type or m-type) were identified as the predominant proteins in the total protein fraction and the UPP peak 1 fraction (125, 315, 319) (Figure 5). In two spots where the predominant protein was a traditional alpha or a gamma gliadin in the total protein fraction, the predominant protein in the UPP peak 1 fraction was a chain-terminating alpha gliadin containing seven cysteines instead of the usual six (346, 546) (Figure 5). One spot identified as triticin in the total protein fraction was identified as a LMW-GS in the UPP peak 1 fraction (348) and one spot identified as a gamma gliadin in the total protein fraction was identified as a triticin in the UPP peak 1 fraction (134).

**Table 1:** Comparison of MS/MS identifications of proteins in 2-DE spots from a total 2% SDS protein extract with those from UPP and EPP fractions

	**Total 2% SDS extract**^**1**^	**UPP peak 1**	**UPP peak 2**	**EPP peak 1**	**EPP peak 2**
**Spot #**^**2**^	**Predominant protein**^**3**^	**Predominant protein**^**4**^	**Predominant protein**^**4**^	**Predominant protein**^**4**^	**Predominant protein**^**4**^
51	HMW-GS Dy10	HMW-GS By9			
124	Alpha gliadin Bu-BQ807130	LMW-GS Bu-3 (s-type)			Alpha gliadin TC11_300663
125	LMW-GS [GenBank: AAB48469] (i-type)	LMW-GS Bu-3 (s-type)			
134	Gamma gliadin Bu-5	Triticin	Gamma gliadin Bu-5		Gamma gliadin Bu-5
161	LMW-GS Bu-3 (s-type)				Alpha gliadin Bu-27
163	Gamma gliadin Bu-11	LMW-GS Bu-8 (m-type)	Alpha gliadin Bu-15	Alpha gliadin TC11_284270^5^	Alpha gliadin Bu-15
				Glyceraldehyde phosphate dehydrogenase	
166	Gamma gliadin Bu-4	LMW-GS Bu-1 (m-type)	Gamma gliadin Bu-4	Gamma gliadin Bu-4	Gamma gliadin Bu-4
169	Gamma gliadin Bu-4	LMW-GS Bu-1 (m-type)	LMW-GS Bu-1 (m-type)	Gamma gliadin Bu-4	Gamma gliadin Bu-3
172	Gamma gliadin Bu-10 or Bu-3	Gamma gliadin Bu-3	Alpha gliadin Bu-9		Alpha gliadin Bu-12
	Alpha gliadin Bu-1 or Bu-3				
	Alpha gliadin Bu-9				
177	Alpha gliadin Bu-BQ806209				Alpha gliadin TC11_315573
310	LMW-GS Bu-3 (s-type)	LMW-GS Bu-20 (s-type)			
315	LMW-GS Bu-11 (m-type)	LMW-GS Bu-2 (s-type)			
316	LMW-GS Bu-3 (s-type)	LMW-GS Bu-2 (s-type)			
318	LMW-GS Bu-2/1-2/-13 (s-type)				LMW-GS Bu-11 (m-type)
319	LMW-GS TC11_277270 (m-type)	LMW-GS Bu-12 (s-type)			
320	Gamma gliadin Bu-5	LMW-GS Bu-2 (s-type)	Gamma gliadin Bu-5		Gamma gliadin Bu-5
		Gamma gliadin Bu-5			
323	Gamma gliadin Bu-5	LMW-GS Bu-2 (s-type)	Gamma gliadin Bu-5		Gamma gliadin Bu-5
324	Gamma gliadin Bu-5	LMW-GS Bu-2 (s-type)	Gamma gliadin Bu-5		Gamma gliadin Bu-5
325	Gamma gliadin Bu-1 or Bu-8	Gamma gliadin Bu-2	Gamma gliadin Bu-1		Gamma gliadin Bu-1
		LMW-GS Bu-7 (m-type)			
327	Alpha gliadin Bu-11	Alpha gliadin TC11_314676 (like alpha Bu-12)	Alpha gliadin Bu-11		Alpha gliadin Bu-11
328	Alpha gliadin Bu-12		Gamma hordein		Gamma hordein
329	Alpha gliadin Bu-12		Alpha gliadin Bu-12	Glyceraldehyde phosphate dehydrogenase	Alpha gliadin Bu-12
330	Alpha gliadin Bu-2	LMW-GS Bu-8 (m-type)	Alpha gliadin Bu-15	Alpha gliadin TC11_284270^5^	Alpha gliadin Bu-12
331	Alpha gliadin Bu-14		Alpha gliadin Bu-4		Alpha gliadin Bu-4
	Alpha gliadin Bu-12				
334	Alpha gliadin Bu-BQ838853		Alpha gliadin Bu-2	Farinin Bu-3	Gamma gliadin Bu-7
	Gamma gliadin-Bu-7				Alpha gliadin Bu-5 or Bu-14
341	Alpha gliadin Bu-23	Alpha gliadin Bu-23	Alpha gliadin Bu-23	Glyceraldehyde phosphate dehydrogenase	Alpha gliadin Bu-8
342	Alpha gliadin Bu-1		Alpha gliadin Bu-1	Gamma gliadin Bu-3	Alpha gliadin Bu-1
343	LMW-GS Bu-8 (m-type)	LMW-GS Bu-1 (m-type)			
		Gamma gliadin Bu-3			
346	Gamma gliadin Bu-6	Alpha gliadin Bu-2	Gamma gliadin Bu-6	Aspartic proteinase	Gamma gliadin Bu-6
348	Triticin	LMW-GS Bu-7 (m-type)			
424	HMW-GS Dy10	HMW-GS By9			
467	Alpha gliadin Bu-4	Alpha gliadin Bu-4	Alpha gliadin Bu-4	LMW-GS Bu-8 (m-type)	Alpha gliadin Bu-4
		LMW-GS Bu-8 (m-type)			
468	Alpha gliadin Bu-3	Alpha gliadin Bu-3	Alpha gliadin Bu-3	Glyceraldehyde phosphate dehydrogenase	Alpha gliadin Bu-3
		LMW-GS Bu-8 (m-type)			
527	Gamma gliadin Bu-11		Alpha gliadin Bu-12		Gamma gliadin Bu-11
546	Alpha gliadin Bu-14	Alpha gliadin Bu-2			
550	Alpha gliadin Bu-10	Alpha gliadin Bu-12		Gamma gliadin Bu-4	Alpha gliadin Bu-12

^1^Data is from Dupont et al., [2].

^2^Spot numbers correspond to those in Dupont et al., [2].

^3^Determined in Dupont et al., [2] on the basis of the number of unique peptides assigned to each protein sequence. Where similar numbers of unique peptides were assigned to more than one protein, multiple identifications are reported.

^4^Based on the number of spectra that were associated with each protein sequence. For each spot the protein with the greatest number of spectra was deemed to be the predominant protein. Where similar numbers of spectra were associated with multiple proteins, more than one identification is reported.

Data is shown only for spots that yielded different proteins in the different fractions.

^5^Contains 7 cysteines.

Twenty-seven spots from the UPP peak 2 fraction were also identified in this study (Additional file 1: Table S1). Two spots identified as LMW-GS in the UPP peak 1 fraction were identified as gamma gliadins in the UPP peak 2 fraction (323, 324) while two spots that contained LMW-GS in the UPP peak 1 fraction were identified as alpha gliadins in the UPP peak 2 fraction (163, 330) (Figure 5, Table 1). A spot containing triticin in the UPP peak 1 fraction was identified as a gamma gliadin in the UPP peak 2 fraction (134) (Figure 5). Two spots in the UPP peak 1 fraction that contained either chain-terminating alpha or gamma gliadins (172, 346) were identified as traditional gliadins without the extra cysteines while one spot identified as a LMW-GS in the UPP peak 1 fraction was identified as a chain-terminating gamma gliadin in the UPP peak 2 fraction (166).

Eighteen spots from the EPP peak 1 fraction were identified (Additional file 1: Table S1). Gliadins containing odd numbers of cysteines were the predominant proteins in five spots in this fraction that contained either LMW-GS (163, 166, 169, 330) or an alpha gliadin (550) in the UPP peak 1 fraction (Figure 5, Table 1). Non-gluten proteins were identified in several spots that contained gluten proteins in other fractions (163, 329, 341, 346, 468).

Nine spots that contained LMW-GS as predominant proteins in the UPP peak 1 fraction were identified as either alpha (124, 163, 330, 468) or gamma (166, 169, 320, 323, 324) gliadins in the EPP peak 2 fraction (Figure 5, Table 1, Additional file 1: Table S1). Spots 166 and 169 contained gamma gliadins with nine cysteines. Two spots identified as chain-terminating gliadins in the UPP peak 1 fraction (172, 346) were identified as other traditional gliadins in the EPP peak 2 fraction. One spot in this fraction also was identified as gamma hordein (328).

### Protein composition of the UPP peak 1 fraction

Because of the relationship between the UPP Peak 1 fraction and flour quality, the individual components of this fraction are of particular interest. Normalized volumes of 2-DE spots in the UPP peak 1 fraction were determined and expressed as a percentage of total spot volume (Additional file 2: Table S2). Using the MS/MS identifications obtained for proteins in this fraction, spot volumes were summed for the various protein types (Table 2). HMW-GS made up 28.5% of the total spot volume in the UPP peak 1 fraction. Overall, the x-type subunits comprised a greater proportion of the HMW-GS (63.3%) than the y-type subunits (36.7%). HMW-GS Bx7 was the most abundant subunit and accounted for 26.5% of the total HMW-GS. Traditional LMW-GS accounted for 44.7% of the total protein in the fraction. LMW-GS are generally classified as m-, s- or i-type based on their N-terminal amino acid sequences. Among the LMW-GS, m-type comprised 51.9% of the LMW-GS while s-type accounted for 41.2% and i-type comprised only 6.9% of the LMW-GS. The ratio of HMW-GS to traditional LMW-GS in the UPP peak 1 fraction was 0.64.

**Table 2:** Protein composition of the UPP peak 1 fraction determined by quantitative 2-DE

**Protein type**	**% Total spot volume**
HMW-GS By9	5.33
HMW-GS Ax2*	4.24
HMW-GS Bx7	7.57
HMW-GS Dx5	6.24
HMW-GS Dy10	5.14
LMW-GS Bu-1 (m-type)	9.11
LMW-GS Bu-2 (s-type)	6.86
LMW-GS Bu-3 (s-type)	9.76
LMW-GS Bu-4 (i-type)	3.08
LMW-GS Bu-6 (m-type)	2.10
LMW-GS Bu-7 (m-type)	7.10
LMW-GS Bu-8 (m-type)	1.97
LMW-GS Bu-12 (m-type)	0.95
LMW-GS Bu-18 (m-type)	1.97
LMW-GS Bu-20 (s-type)	1.82
Chain-terminating alpha gliadins^1^	1.18
Chain-terminating gamma gliadins^2^	1.20
Chain-terminating omega gliadins^3^	3.05
Alpha gliadins	7.44
Gamma gliadins	2.36
Omega gliadins	2.81
Serpins	3.41
Triticins	3.84
Globulins	0.57

^1^Includes spot numbers 338, 346, 546.

^2^Includes spot numbers 172, 337.

^3^Includes spot numbers 107, 113, 115, 116.

Because alpha, gamma and omega gliadins containing extra cysteines could be distinguished by MS/MS from traditional gliadins, it was also possible to determine the proportions of chain-terminating gliadins in this fraction (Table 2). Three spots identified as alpha gliadin Bu-2, a protein containing seven cysteines (338, 346, 546), comprised 1.2% of the total spot volume. One spot identified as gamma gliadin Bu-3 (172) and one spot identified as gamma gliadin Bu-4 (337), both containing nine cysteines, together comprised 1.2% of the total spot volume. Four spots identified as omega gliadins containing single cysteines (107, 113, 115, 116) accounted for 3.1% of the total spot volume. Including the chain-terminators, the glutenins made up 78.6% of the total protein in the UPP peak 1 fraction.

Monomeric gliadins comprised only 12.6% of the UPP peak 1 fraction with alpha gliadins accounting for the largest share (7.4% of total) and gamma and omega gliadins accounting for only 2.4 and 2.8%, respectively (Table 2). The ratio of gliadins to glutenins in the UPP peak 1 fraction was therefore 0.16. Several types of non-gluten proteins also were accumulated in the UPP peak 1 fraction. Serpins comprised 3.4%, triticins accounted for 3.8%, and globulins were responsible for 0.6% of the total spot volume of the fraction.

### Partitioning of proteins in other fractions

While components of the UPP peak 1 fraction may be important for flour quality, a better understanding of how the wheat flour proteins partition into the various fractions may provide new insights into the nature of the glutenin polymer. Table 3 shows the relative proportions of spots that contained single protein types (HMW-GS, omega gliadins, serpins, triticins, globulins) or were identified in this study (chain-terminating gliadins) in the UPP and EPP peak 1 and peak 2 fractions. The proportions of alpha gliadins, gamma gliadins and LMW-GS are not reported because the amounts of these proteins could not be quantified without first confirming the identities of all protein spots from each fraction. The HMW-GS were found primarily in peak 1 of both the UPP and the EPP fractions, comprising 28.5 and 20.2% of the protein in these fractions, respectively. In both fractions, there was a greater proportion of x-type HMW-GS than y-type HMW-GS and the Bx7 HMW-GS subunits were present in the greatest amounts. The HMW-GS comprised only a small portion of the peak 2 fractions from both the UPP and the EPP samples, 4.8% and 10.4%, respectively, and Dx5 was the most prevalent subunit. In contrast, the omega gliadins were very abundant in peak 2 of both the UPP and EPP fractions, comprising 20.1 and 16.3% of the total protein, respectively, but accounted for only a very small proportion of the peak 1 fractions.

**Table 3:** Summary of protein types found in other protein fractions

	**% Total spot volume**		**% Total spot volume**	
	**PEAK 1**		**PEAK 2**	
	**UPP**	**EPP**	**UPP**	**EPP**
HMW-GS By9	5.33	3.64	0.49	1.73
HMW-GS Ax2*	4.24	3.83	0.75	1.47
HMW-GS Bx7	7.57	5.77	0.80	2.58
HMW-GS Dx5	6.24	3.63	1.95	2.74
HMW-GS Dy10	5.14	3.31	0.77	1.87
Total HMW-GS	28.51	20.18	4.76	10.38
Total omega gliadins	2.81	3.73	20.08	16.25
Chain-terminating alpha gliadins	1.18	2.67^1^	1.60^4^	0.32^6^
Chain-terminating gamma gliadins	1.20	8.63^2^	1.10^5^	4.24^7^
Chain-terminating omega gliadins	3.05	3.37	2.59	2.53
Total chain-terminating gliadins	5.43	14.67	5.28	7.09
Serpins	3.41	5.74	1.03	1.01
Triticins	3.84	7.21^3^	2.61^3^	2.57^3^
Globulins	0.57	2.55	0.59	0.99

^1^Includes spots 163, 330, 338.

^2^Includes spots 166, 169, 337, 342, 550.

^3^Includes spots 136, 143, 249, 253, 423, 463.

^4^Includes spot 334.

^5^Includes spots 166, 337.

^6^Includes spot 338.

^7^Includes spots 166, 169, 337.

Chain-terminating gliadins were distributed among all the protein fractions but made up a larger proportion of the EPP peak 1 fraction than the other protein fractions, 14.7% of total protein (Table 3). The bulk of the chain terminators were represented by gamma gliadins Bu-3 and Bu-4. These accounted for 8.6% of the total protein in the EPP peak 1 fraction, 4.2% in the EPP peak 2 fraction and 1.2% or less in the other fractions. Chain-terminating alpha gliadins also were more predominant in the EPP peak 1 fraction while omega gliadins comprised similar proportions of the different fractions.

The EPP peak 1 fraction also contained a higher proportion of several types of non-gluten proteins (Table 3). Globulins comprised 2.6% of the protein in the EPP peak 1 fraction but only 0.6 to 1% of the protein in the other fractions. Triticins accounted for 7.2% of the protein in the EPP peak 1 fraction but less than 3.8% in the other fractions. Serpins comprised larger proportions of the peak 1 fractions than the peak 2 fractions, 5.7% of the protein in the EPP peak 1 fraction, 3.4% in the UPP peak 1 fraction and only 1% of the UPP and EPP peak 2 fractions. All together, these non-gluten proteins accounted for 15.5% of the total protein in the EPP peak 1 fraction.

## Discussion

Although the 2-DE analysis revealed that the HMW-GS and LMW-GS generally partitioned into peak 1 (polymer) fractions and the gliadins partitioned into peak 2 (monomer) fractions, it was apparent that many 2-DE spots were present in multiple fractions. MS/MS analysis of the different fractions revealed that spots with the same pI and MW coordinates often yielded different identifications, clearly demonstrating that there are many layers of complexity to the wheat flour proteome that should be considered in evaluating future experiments. The analysis yielded a more precise picture of the protein composition of the polymer fraction most associated with flour quality (UPP peak 1) as well as new information about the composition of the fraction containing smaller glutenin polymers (EPP peak 1).

Because of the excellent MS/MS sequence coverage, gliadins containing an odd number of cysteine residues were distinguished from monomeric alpha, gamma and omega gliadins containing either six, eight or no cysteine residues, respectively. It has been hypothesized that these proteins are incorporated into the polymer and serve as chain terminators, thereby reducing polymer size [5]. A number of studies have reported the presence of proteins with gliadin-like sequences in glutenin polymer fractions [16-18]. This study demonstrates not only the presence of gliadins containing an odd number of cysteines in the polymer fraction but also their accumulation in the fraction containing extractable polymers (EPP peak 1), thereby supporting their role as chain-terminators. It is curious that gamma gliadins Bu-3 and Bu-4 containing nine cysteines were present at higher proportions than the other chain-terminating alpha and omega gliadins in the EPP peak 1 fraction from Butte 86 flour. It would be interesting to determine whether the accumulation of these gamma gliadins influences the overall amount of polymeric protein that partitions into the 0.5% SDS extractable fraction (EPP peak 1). Gene silencing experiments specifically targeting gamma gliadins Bu-3 and Bu-4 in Butte 86 could provide insight into the roles of these proteins in the formation of the glutenin polymer.

Several groups of non-gluten proteins also comprised a greater percentage of the protein in the EPP peak 1 fraction than in the other fractions. These included triticins, globulins and serpins. Triticins and globulins are similar to 11S and 7S storage proteins from dicots that are known to form trimeric and hexameric structures [1] so it might be expected that these proteins would be found in the extractable polymer fraction. The prevalence of serpins in this fraction is perhaps more surprising. All of the 13 spots identified as serpins comprised a larger percentage of the protein in the EPP peak 1 fraction than the other fractions. It has been reported previously that about 40% of the total serpin can be extracted from flour with aqueous solutions. The remaining serpins are “bound” and require extraction with DTT [19], suggesting that there are either interactions between individual serpins or between serpins and gluten proteins. The complement of serpin sequences is incomplete in Butte 86 [2]. However, a survey of wheat sequences from NCBI revealed serpins containing either one, two or three cysteine residues. This raises the possibility that certain serpins may be covalently linked into the glutenin polymer and might serve as chain terminators, analogous to the chain-terminating gliadins. Further studies are warranted on this group of proteins and possible links to flour quality.

The current study provides a foundation for further investigations into how agronomic and environmental factors might influence glutenin polymer distribution. In previous studies, MacRitchie and Gupta [20] found that sulfur deficiency increased the percent of unextractable polymeric protein and the ratio of HMW-GS to LMW-GS while Irmak [21] reported that high temperature decreased the percentage of unextractable polymeric protein in mature grain. Our group has noted variations in the levels of specific flour proteins in response to fertilizer [22] and high temperature [23,24] using quantitative 2-DE. It is intriguing that serpins were among the proteins shown to change in amount in response to both fertilizer [25] and high temperature [24] given their abundance in the EPP peak 1 fraction. Further studies of the glutenin polymer fractions using 2-DE combined with MS/MS should provide insight into how growth conditions of the plant influence glutenin polymer and flour quality.

## Methods

### Protein preparation

Plants of the hard red spring wheat *Triticum aestivum* L. cv. Butte 86, were grown in a climate-controlled greenhouse under a 24°C/17°C day/night regimen and grain was milled to flour as described previously [26,27]. Wheat flour protein fractions were prepared by the method of Gupta and MacRitchie [12] (Figure 2). For the total protein fraction, 10 mg of flour was suspended in 1 ml of 0.5% SDS buffer (0.5% SDS, 50 mM Na phosphate buffer, pH 6.9) and the sample sonicated for 10 seconds at 35% attenuation (Sonics Vibra Cell sonicator fitted with a 3 mm VCX130 Probe, Sonic and Materials Inc., Newtown, CT). Following sonication, the sample was centrifuged at 15,900 *g* for 20 min (Eppendorf Centrifuge 5418, Brinkman Instruments, Inc., Westbury, NY) and the supernate retained. For the SDS-extractable and unextractable fractions, 10 mg of flour was suspended in 1 ml of 0.5% SDS and incubated for five minutes at room temperature on a platform rocker (Low Profile Rocker, Stovall Life Science, Inc., Greensboro, NC). The sample was centrifuged at 15,900 *g* for 20 min and the supernate (0.5% SDS extractable polymeric protein, EPP) retained. The pellet was suspended in 1 ml 0.5% SDS buffer and sonicated for 20 seconds. Following sonication, the sample was centrifuged at 15,900 *g* for 20 min and the supernate (0.5% SDS unextractable polymeric protein, UPP) retained. Three separate samples of wheat flour were extracted.

### Fractionation of proteins by SEC

EPP and UPP fractions were filtered through 0.45 μm filters (Ultrafree-MC, centrifugal filters, PVDF, Millipore Corp., Billerica, MA) by centrifugation at 16,900 *g* for 10 min (Eppendorf 5418). SEC was carried out using a Hewlett Packard Series II 1090 liquid chromatograph (Santa Clara, CA) fitted with a BioSep-SEC-s4000 column (7.8 × 300 mm, 5 μ particle dia, 500 Å pore size, Phenomenex, Torrance, CA) and a diode array detector set to monitor 210 nm. Data were acquired with a PC work station (Chem Station for Liquid Chromatography 3D System, Agilent Technologies, Santa Clara, CA). EPP and UPP (100 μL) were injected manually and proteins eluted at 0.5 ml/min with 50% acetonitrile/water/0.05% TFA. Three fractions were collected for each sample corresponding to peak 1: 9.6 to 14.3 min, ~2.4 ml; peak 2: 14.3 to 18 min, ~1.9 ml; peak 3: 18-20.5 min, ~1.3 ml. Fractions from 5 injections were pooled, stored at -20°C overnight and then vacuum dried (Speed Vac SC110, Savant Instruments, Farmingdale, NY). The protein amount in each fraction was determined by the method of Lowry [28] as modified by Hurkman and Tanaka [29].

### Quantitative 2-DE analysis

The total protein fraction was precipitated with TCA by the method of Sanchez [ http://www.its.caltech.edu/~bjorker/Protocols/TCA_ppt_protocol.pdf] to remove SDS and salts that interfere with isoelectric focusing (IEF). One volume of 6.1 N TCA was added to 4 volumes of sample, and incubated on ice for 10 min. Samples were centrifuged for 15 min at 11,600 *g* and 4°C (TOMY MRX-151 High Speed Micro Refrigerated Centrifuge, Peninsula Labs, Inc., Belmont, CA) and the supernate discarded. The pellets were rinsed 3 times with 200 μl cold (-20°C) acetone. The pellet suspensions were centrifuged at 11,600 *g* at 4°C for 10 min after each rinse and the final pellet was air dried at room temperature. The protein amount in the total protein fraction was determined by the method of Lowry [28]. Urea buffer (9 M urea, 4% Nonidet P-40, 1% DTT, and 2% 3-10 Iso-Dalt Grade Servalyts) was added to the dried pellets of the total protein fraction and the EPP and UPP SEC fractions. Samples were incubated at room temperature for 1 hr in a microtube shaker (TOMY Micro Tube Mixer MT-360, Peninsula Labs, Inc., Belmont, CA) and were then centrifuged at 16,900 *g* for 10 min at room temperature and the supernates retained.

Proteins were separated by 2-DE as described previously [2,29]. IEF was performed using a Mini Protean II Tube Cell (BioRad Laboratories, Richmond, CA). The first dimension capillary tube gels contained 9.2 M urea, 4% (total monomer) acrylamide:BIS, 2% Nonidet P-40, 2% 3-10 Iso-Dalt Grade Servalyts, 0.015% ammonium persulfate, and 0.125% TEMED. The upper electrode (anode) buffer was 0.2% (v/v) sulfuric acid and the lower electrode buffer (cathode) was 0.5% (v/v) ethanolamine. Because the anode buffer was acidic, the wires from the electrophoresis cell were reversed at the power supply. The gels were pre-focused at 200 V for 10 min, 300 V for 15 min and 400 V for 15 min. Fifteen μg protein of the total protein fraction, EPP peak 1, EPP peak 2, EPP peak 3, UPP peak 1, UPP peak 2, and UPP peak 3 were loaded onto the IEF gels and overlain with 5 M urea. IEF gels were run at 500 V for 10 min and then increased to 750 V for 1 h. Gels were extruded into tubes containing equilibration buffer (2.3% SDS, 10% glycerol, 0.05% dithiothreitol, 62.5 mM Tris-HCl pH 6.8). Gels were frozen immediately on dry ice and stored at -80°C. Proteins were separated in the second dimension by SDS gel electrophoresis using an XCell SureLock Mini-Cell electrophoresis system (Life Technologies, Grand Island, NY). IEF gels were thawed and immediately placed on top of Novex NuPage 4-12% acrylamide Bis-Tris precast gels, 1 mm thick with 2-D well (Life Technologies). The IEF gel was overlain with 45 μl of equilibration buffer. Four μl of Novex Sharp Protein Standard (Life Technologies) were loaded into the standard well. The SDS gels were run with NuPAGE MES SDS running buffer (Life Technologies) for 50 min at 200 V. Gels were stained overnight with Coomassie G-250 as reported in Kasarda et al. [30], destained in water for 2 h at room temperature, and stored at 4°C in 20% ammonium sulfate.

The experimental design included three biological replicates, each with two technical replicates. All 2-DE gels were digitized with a calibrated scanner (Epson Perfection V750 PRO, Long Beach, CA) at 310 dpi. The protein spot pattern of two replicate 2-DE gels of each fraction (EPP peak 1, EPP peak 2, UPP peak 1, UPP peak 2) from three separate flour extractions were aligned and matched to the protein spot pattern of the total protein extract using Progenesis SameSpots Ver. 4.5 (Nonlinear Dynamics Limited, Newcastle upon Tyne, UK). Spot volumes of flour proteins previously identified by tandem mass spectrometry [2] were determined by the Progenesis software for each gel. The replicate gels are displayed in Additional file 3: Figure S1 and spot volumes are reported along with statistical analyses in Additional file 2: Table S2.

### Mass spectrometry

Three sets of selected spots from the 2-D gels were excised for digestion with chymotrypsin, thermolysin or trypsin as reported in Vensel et al. [14]. The excised spots were placed in 96 well plates where they were reduced, alkylated and digested and the resulting peptides collected into a 96 well plate of a DigestPro (Intavis, Koeln, Germany).

Plate containing peptides from the digested gel spots were placed in the autosampler of an EASY-nLC II that was interfaced by nano-electrospray source to an Orbitrap Elite mass spectrometer (Thermo Scientific, San Jose, CA). Ten μl fractions were loaded by the autosampler onto an EASY-column trap (2 cm, ID 100 μm, 5 μm, 120 Å, ReproSil-Pur C18-AQ) that was washed with solvent A to remove salts and was then switched in-line with a 10 cm, ID 75 μm, 3 μm, 120 Å, ReproSil-Pur C18-AQ reverse phase column and eluted with a gradient of acetonitrile into the mass spectrometer. Solvent A was 5% in acetonitrile and Solvent B was 80% in acetonitrile, both solvents were 0.05% in formic acid. Gradient elution was at a flow rate of 250 nl per minute from 100% Solvent A to 35% Solvent B in 45 minutes. Peptides were detected in the Orbitrap with the FT survey scan set to scan a range from 300 to 4000 m/z at a resolution of 60,000. The 10 most intense peaks were subject to collision-induced dissociation (CID). The mass range for the CID scans was set to High and the minimal signal threshold to 10,000. Dynamic exclusion with a repeat count of 2 was enabled for duration of 10 seconds. Normalized collision energy was set to 30%. Mass accuracy of the Orbitrap Elite mass spectrometer is estimated to be at least 10-fold greater than that achieved with the QSTAR Pulsar i quadrupole time-of-flight mass spectrometer used in Dupont et al [2].

### Data analysis

Raw files were converted to MGF files using MSConvert from the ProteoWizard open-source project [31] at [ http://proteowizard.sourceforge.net/downloads.shtml]. The first pass search of the MS/MS spectra was against the Super_Wheat database [2] that contained ~2.1 million plant protein sequences. The first pass database did not have a set of sequences designated as “decoy” sequences but as it was largely constructed from nucleic acid sequences translated in six reading frames there were considerably more “decoy” sequences than “correct” sequences. Version 2.3 of Mascot [ http://www.matrixscience.com/] and X! Tandem version 2012.10.1.2 [ http://www.thegpm.org/tandem/] were used to match instrument spectra to *in silico*-generated spectra. The six analysis files from the searches for each spot (two search engines and three enzymatic digests) were combined in spot-specific folders. Data in individual folders were then analyzed, validated and displayed using Scaffold version 4.07 [ http://www.proteomesoftware.com]. This was named the First Pass search and a subset database of fasta files was generated from it by exporting from Scaffold all proteins that had a protein probability of 20% and a peptide probability of 0%. The subset database contained 2162 sequences. A reverse database was created from the subset database and appended to it, creating a database containing 4,342 sequences that was used for the Second Pass search. The Second Pass search was carried out as for the First Pass search by specifying that each folder was a MUDPIT type experiment with experiment-wide sample grouping. Criteria for the Second Pass search protein acceptance was stringent with filtering set in the Scaffold validation software package to a protein probability of 99% and a requirement for 4 matching peptides having a calculated 95% probability.

## Abbreviations

1-DE: One-dimensional gel electrophoresis; 2-DE: Two-dimensional gel electrophoresis; EPP: Extractable polymeric protein (0.5% SDS soluble protein fraction); HMW-GS: High molecular weight glutenin subunits; IEF: Isoelectric focusing; LMW-GS: Low molecular weight glutenin subunits; SEC: Size-exclusion chromatography; SDS: Sodium dodecyl sulfate; TCA: Trichloroacetic acid; TFA: Trifluoroacetic acid; UPP: Unextractable polymeric protein (sonicated, 0.5% SDS soluble protein fraction).

## Competing interests

The authors declare that they have no competing interests.

## Authors’ contributions

WHV conceived of the project, carried out the mass spectrometry, analyzed the mass spectrometry data and wrote a draft of the manuscript. CKT carried out the gluten protein preparation, SEC, 2-DE analysis and interpretation of the 2-DE data. SBA analyzed and interpreted MS/MS and 2-DE data, wrote portions of the manuscript and provided conceptual framework. All authors read and approved of the final manuscript.

## Supplementary Material

Additional file 1: Table S1 Comparison of MS/MS identifications of proteins in 2-DE spots from a total 2% SDS protein extract with those from UPP and EPP fractions. All proteins identified in each spot from the total protein extract are shown. For the UPP and EPP fractions, only the predominant proteins are shown. Click here for file

Additional file 2: Table S2 Statistical analysis of average normalized volumes of 2-DE spots from UPP and EPP fractions. Values in columns 1, 2 and 3 were obtained from three separate extractions of flour protein. Each value represents the average normalized volume of the spot from 2 replicate gels. Proteins were grouped according to identifications determined from the UPP peak 1 fraction and will be different for the UPP peak 2 fraction and EPP fractions. Click here for file

Additional file 3: Figure S1 Replicate 2-D gels of fractionated flour proteins used for quantitative analysis. Click here for file
