# Supplementary material for: Protein composition of wheat gluten polymer fractions determined by quantitative two-dimensional gel electrophoresis and tandem mass spectrometry
Source: Proteome Sci. 2014 Feb 11;12:8. doi: 10.1186/1477-5956-12-8 (PMC4016294; doi:10.1186/1477-5956-12-8)
Supplement: Additional file 3: Figure S1 — Replicate 2-D gels of fractionated flour proteins used for quantitative analysis. [file 1477-5956-12-8-S3.ppt]

## Slide 1
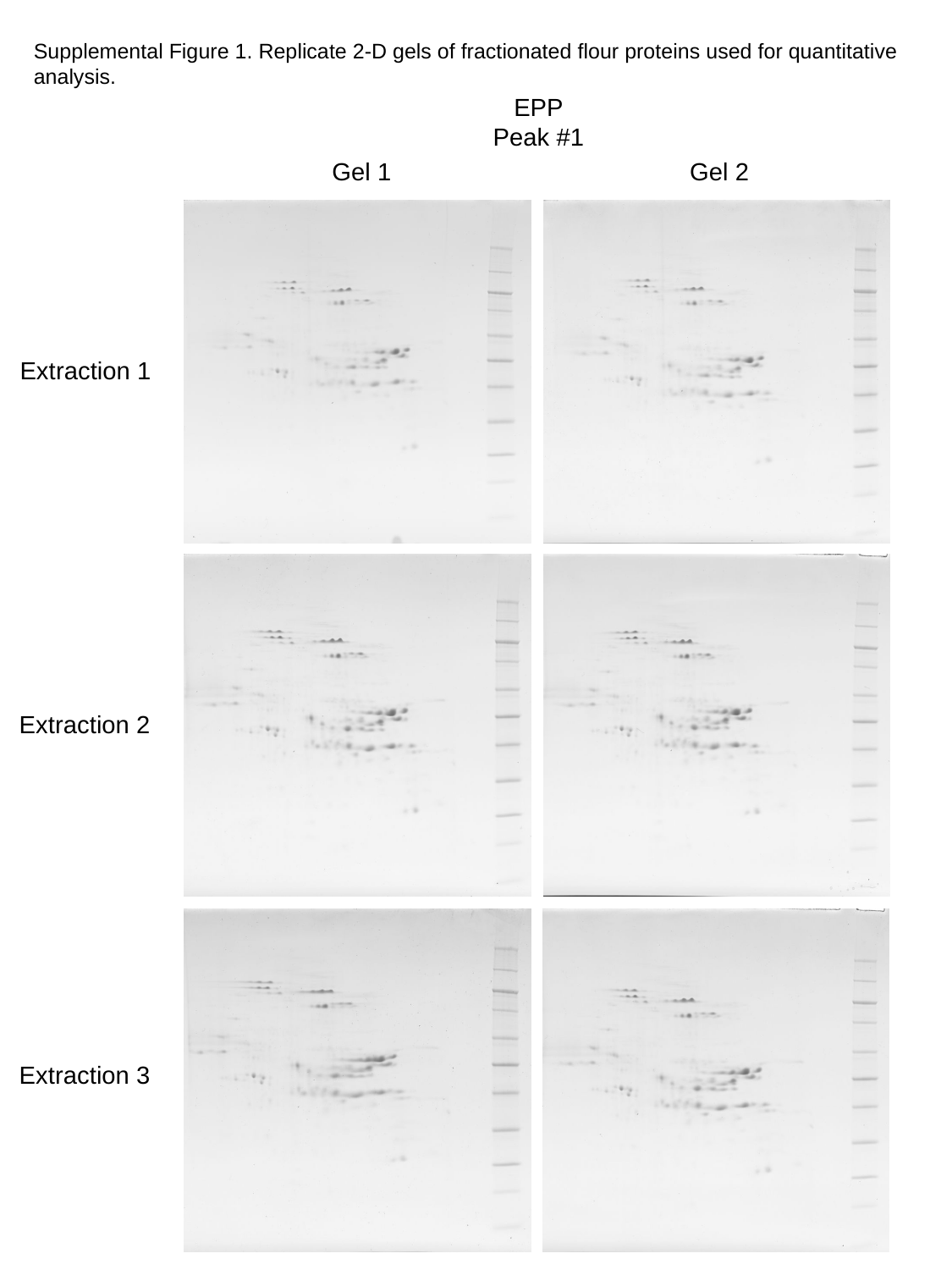

Supplemental Figure 1. Replicate 2-D gels of fractionated flour proteins used for quantitative analysis.
EPP
Peak #1
Gel 1
Gel 2
Extraction 1
Extraction 2
Extraction 3

## Slide 2
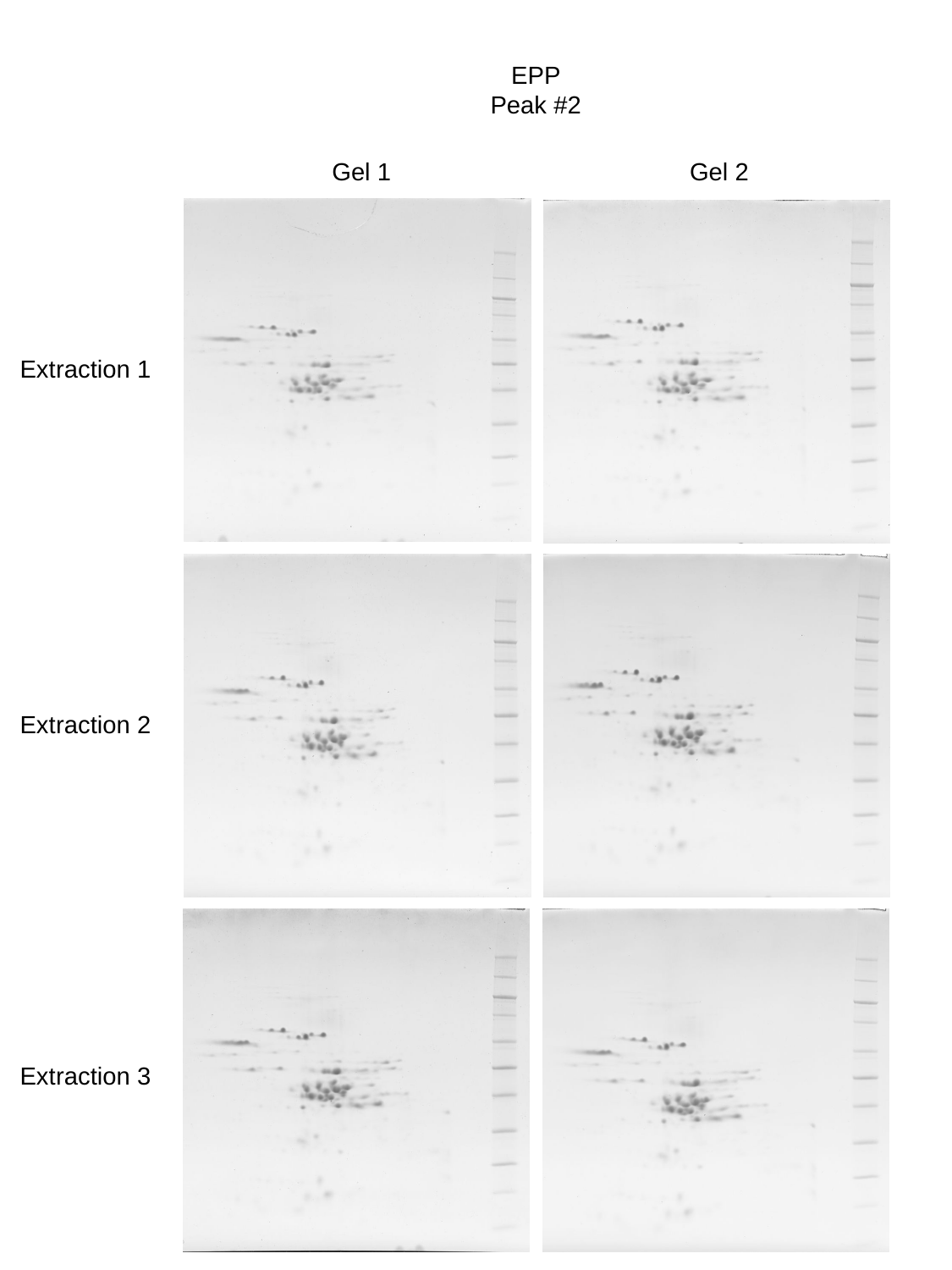

EPP
Peak #2
Gel 1
Gel 2
Extraction 1
Extraction 2
Extraction 3

## Slide 3
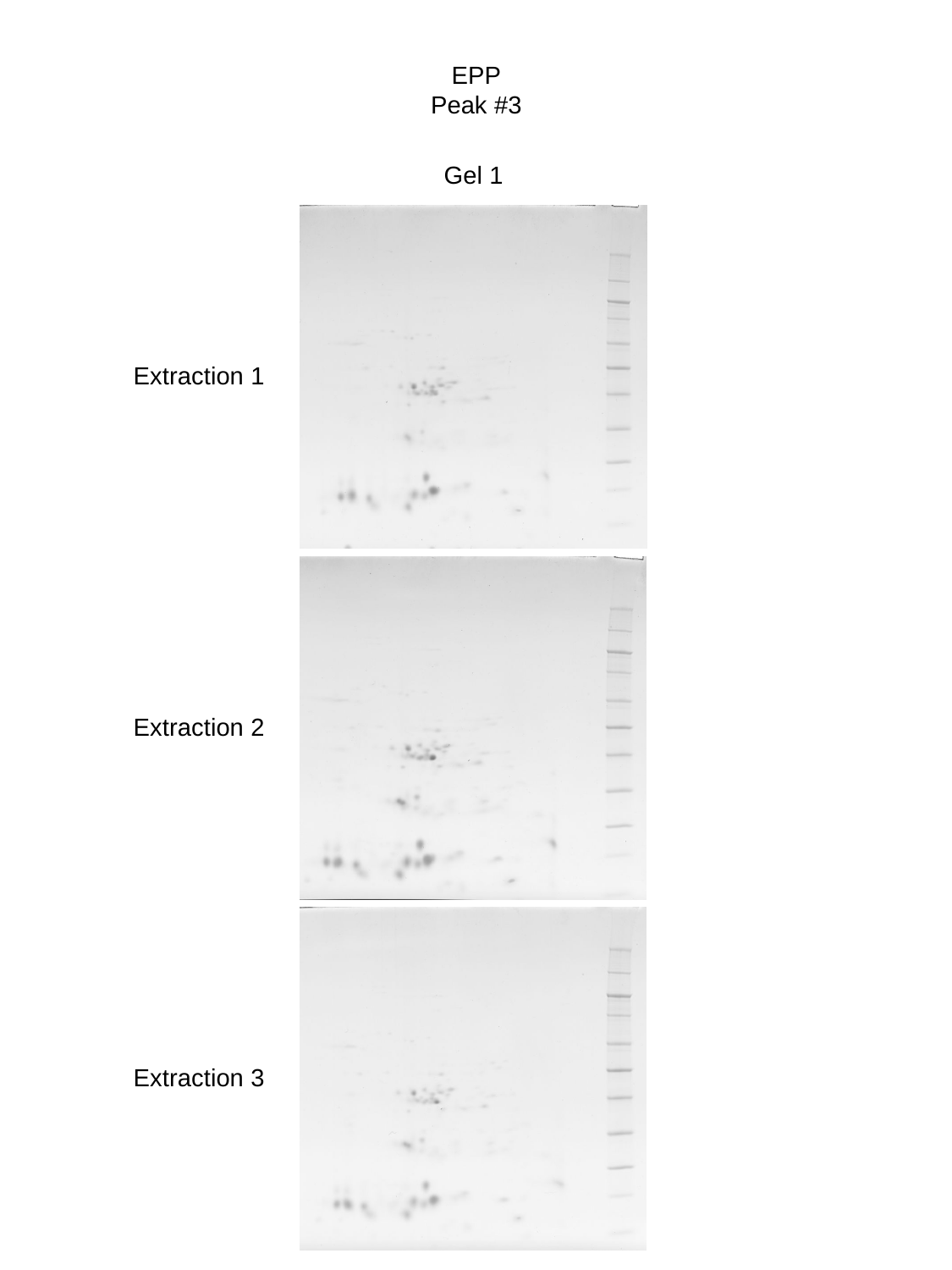

EPP
Peak #3
Gel 1
Extraction 1
Extraction 2
Extraction 3

## Slide 4
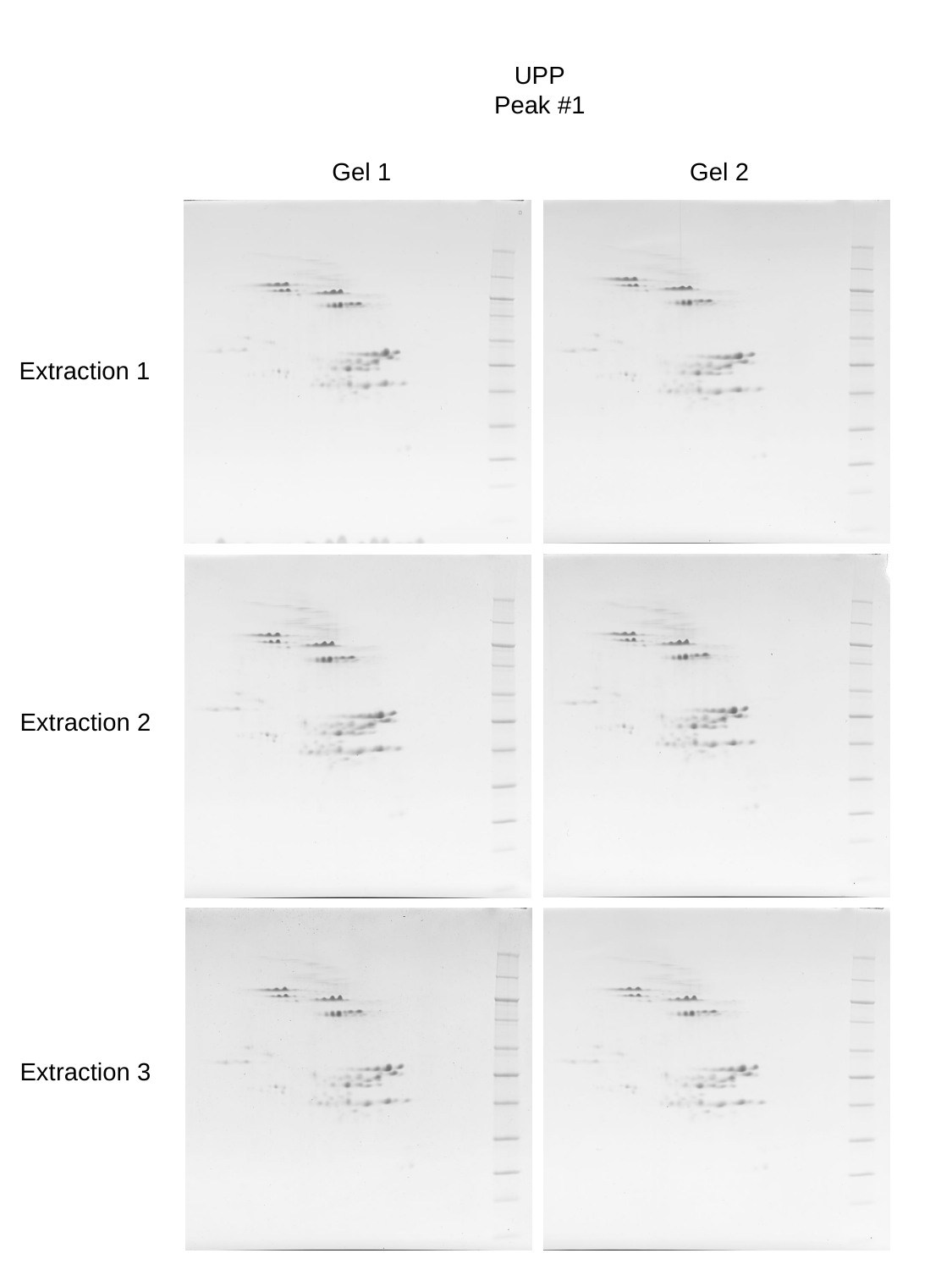

UPP
Peak #1
Gel 1
Gel 2
Extraction 1
Extraction 2
Extraction 3

## Slide 5
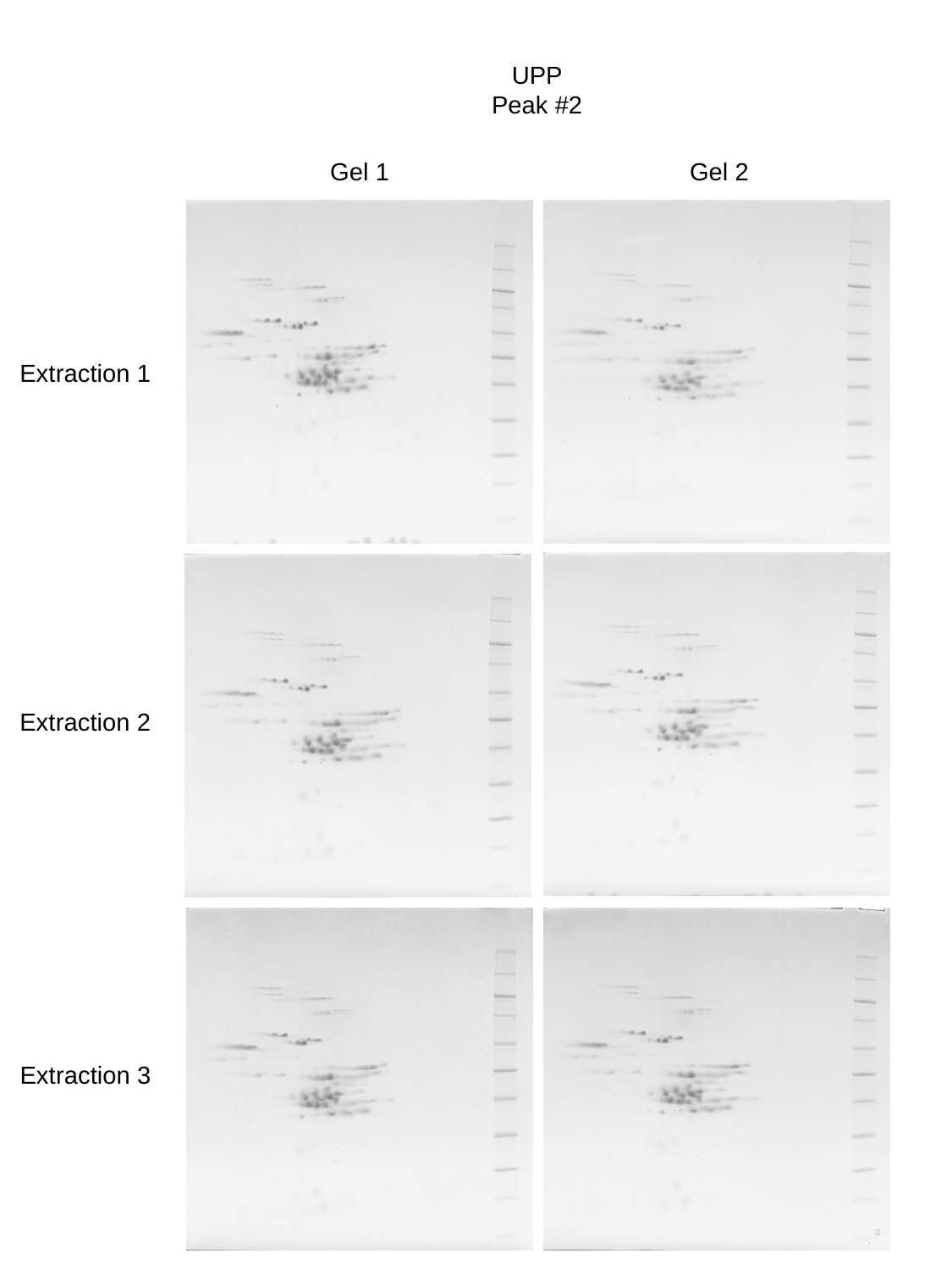

UPP
Peak #2
Gel 1
Gel 2
Extraction 1
Extraction 2
Extraction 3

## Slide 6
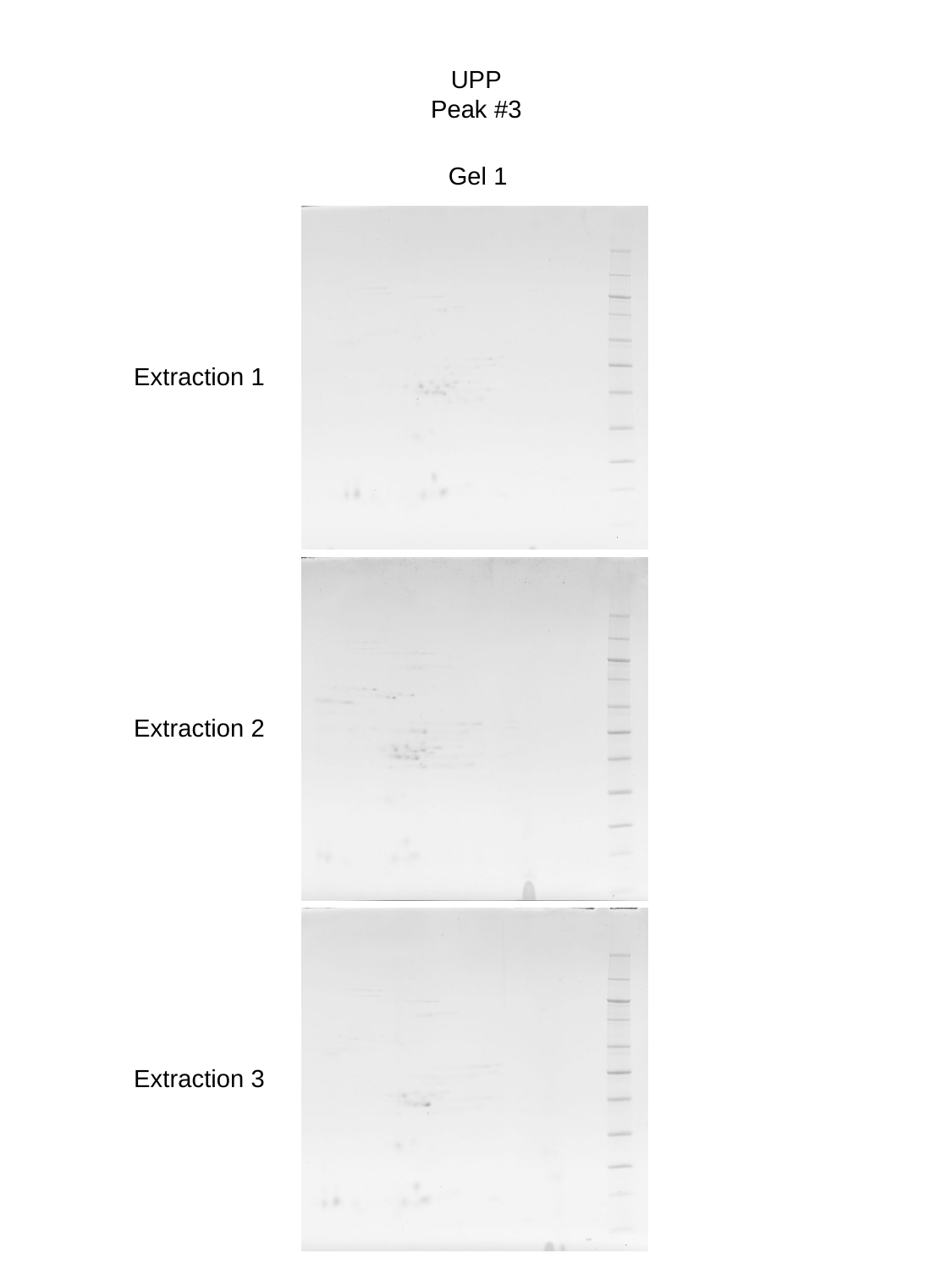

UPP
Peak #3
Gel 1
Extraction 1
Extraction 2
Extraction 3
